# *Cyp2j5*-Gene Deletion Affects on Acetylcholine and Adenosine-Induced Relaxation in Mice: Role of Angiotensin-II and CYP-Epoxygenase Inhibitor

**DOI:** 10.3389/fphar.2020.00027

**Published:** 2020-02-05

**Authors:** Stephanie Agba, Ahmad Hanif, Matthew L. Edin, Darryl C. Zeldin, Mohammed A. Nayeem

**Affiliations:** ^1^Pharmaceutical Sciences, School of Pharmacy, Center for Basic and Translational Stroke Research, West Virginia University, Morgantown, WV, United States; ^2^Division of Intramural Research, NIEHS/NIH, Durham, NC, United States

**Keywords:** CYP-epoxygenases, acetylcholine, adenosine, adenosine A_2A_ receptor, angiotensin-II, relaxation

## Abstract

Previously, we showed vascular endothelial overexpression of human-CYP2J2 enhances coronary reactive hyperemia in Tie2-CYP2J2 Tr mice, and eNOS^−/−^ mice had overexpression of CYP2J-epoxygenase with adenosine A_2A_ receptor-induced enhance relaxation, but we did not see the response in CYP2J-epoxygenase knockout mice. Therefore, we hypothesized that *Cyp2j5*-gene deletion affects acetylcholine- and 5'-N-ethylcarboxamidoadenosine (NECA) (adenosine)-induced relaxation and their response is partially inhibited by angiotensin-II (Ang-II) in mice. Acetylcholine (Ach)-induced response was tested with *N*-(methylsulfonyl)-2-(2-propynyloxy)-benzenehexanamide (MS-PPOH, CYP-epoxygenase inhibitor; 10^−5^M) and Ang-II (10^−6^M). In *Cyp2j5^−/−^* mice, ACh-induced relaxation was different from C57Bl/6 mice, at 10^−5^ M (76.1 ± 3.3 *vs.* 58.3 ± 5.2, *P* < 0.05). However, ACh-induced relaxation was not blocked by MS-PPOH in *Cyp2j5^−/−^*: 58.5 ± 5.0%, *P* > 0.05, but blocked in C57Bl/6: 52.3 ± 7.5%, *P* < 0.05, and Ang-II reduces ACh-induced relaxation in both *Cyp2j5^−/−^* and C57Bl/6 mice (38.8 ± 3.9% and 45.9 ± 7.8, *P <*0.05). In addition, NECA-induced response was tested with Ang-II. In *Cyp2j5^−/−^* mice, NECA-induced response was not different from C57Bl/6 mice at 10^−5^M (23.1 ± 2.1 *vs.* 21.1 ± 3.8, *P* > 0.05). However, NECA-induced response was reduced by Ang-II in both *Cyp2j5^−/−^* and C57Bl/6 mice (−10.8 ± 2.3% and 3.2 ± 2.7, *P <* 0.05). Data suggest that ACh-induced relaxation in *Cyp2j5^−/−^* mice depends on nitric oxide (NO) but not CYP-epoxygenases, and the NECA-induced different response in male *vs.* female *Cyp2j5^−/−^* mice when Ang-II treated.

## Introduction

Arachidonic acid (AA) can be metabolized into epoxyeicosatrienoic acids (EETs) through cytochrome P450 (CYP)-epoxygenases, like *CYP2Cs* and *CYP2Js*, and the cytochrome P450 (CYP) ω-hydroxylases (*CYP4A*, *CYP4B*, *CYP4F*, *CYP4V*, *CYP4X*, *CYP4Z*, etc.) hydroxylate AA to HETEs (hydroxyeicosatetraenoic acids), including 20-HETE which is a potent vasoconstrictor (Hoopes et al., 2015). *CYP2C* and CYP2J-epoxygenases generate four distinct EET regioisomers: 5,6-, 8,9-, 11,12-, and 14,15-EET, and these EETs are involved in numerous biological functions, such as hyperpolarization and relaxation in vascular smooth muscle cells (Campbell et al., 1996; Ellinsworth et al., 2014). Further, several members of *CYP2C* (*Cyp2c29*) and *CYP2J* (*CYP2J2*, *Cyp2j5*) subfamilies have been detected on the vascular endothelium (Fisslthaler et al., 1999; Node et al., 1999; Zeldin and Liao, 2000; Zeldin et al., 2001; Nayeem et al., 2008; Nayeem et al., 2009; Nayeem et al., 2011; Ponnoth et al., 2012a; Pradhan et al., 2014; Hanif et al., 2017b). In addition, Yang et al., and Hanif et al., reported that an overexpression of CYP2J2 protects vascular endothelium against hypoxia-reoxygenation injury/ischemia/reperfusion injury with enhanced coronary reactive hyperemic (CRH) response in isolated mouse heart model (Yang et al., 2001; Hanif et al., 2017b), and the vascular endothelial CYP2Cs and CYP2Js are the main source of EETs (5,6-, 8,9-, 11,12-, and 14,15-EET regioisomers) generation (Rosolowsky and Campbell, 1996; Fisslthaler et al., 1999; Node et al., 1999). EETs have shown to be more effective on small-resistance vessels, as well as in coronary arteries and aorta (Fang et al., 2001; Nayeem et al., 2008; Nayeem et al., 2013; Hanif et al., 2016a; Hanif et al., 2016b; Hanif et al., 2017b). EETs have been observed to produce relaxation in isolated coronary arteries at concentrations as low as 10 pM and are involved in increased CRH response (Fang et al., 2001; Hanif et al., 2016a; Hanif et al., 2016b; Hanif et al., 2017b). In addition, the EET-induced relaxation in bovine coronary arteries and mouse aorta is inhibited by the EET antagonist, 14,15-epoxyeicosa-5(*Z*)-enoic acid (Gauthier et al., 2002; Nayeem et al., 2009; Nayeem et al., 2013; Pradhan et al., 2014).

EETs dilate blood vessels and also have natriuretic effects (Oltman et al., 1998; Imig et al., 2001; McGiff and Quilley, 2001), and the renal CYP-epoxygenases (CYP2C and CYP2J subfamilies) are believed to be under the regulatory control of dietary salt (Makita et al., 1994). Therefore, spontaneously hypertensive rats (SHR) have shown altered protein expression levels and activities for both renal CYP-epoxygenase and ω-hydroxylase (Schwartzman et al., 1996; Yu et al., 2000). Athirakul et al. reported that *Cyp2j5* gene disruption resulted in increased blood pressure in female mice compared to their wild-type counterparts (Athirakul et al., 2008), and the elevated blood pressure was associated with increased left ventricular mass and enhanced renal (afferent arterioles) vasoconstriction due to angiotensin II (Athirakul et al., 2008). Further, an increase in renal vascular reactivity to angiotensin II has been reported in the early stage of hypertension in both rodents and humans (Simon et al., 1995; Imig, 2000). *Cyp2j5* enzyme is abundant in kidney as well as in the mouse aorta where it is very active in arachidonic acid metabolism to generate EETs (Ma et al., 2004; Nayeem et al., 2008; Nayeem et al., 2013). Mouse kidneys demonstrated higher *Cyp2j5* expression in male compared to female mice after puberty, and Northern analysis also revealed that *Cyp2j5* transcripts were more abundant in adult male *versus* adult female kidneys (Ma et al., 2004). Burgess *et al*. showed that *Cyp2j5* is responsible for production of primarily 14,15- and 11,12-EETs in visceral adipose tissue (Burgess et al., 2012), and overexpression of *Cyp2j5* and *Cyp4a* proteins were observed in *Ephx2*^−/−^ *vs.* C57Bl/6 mice with enhanced adenosine (NECA) and CGS 21680 (A_2A_ AR)-induced aortic relaxation (Nayeem et al., 2013). An overexpression of *Cyp2j5* and *Cyp4a* proteins in *Ephx2*^−/−^ *vs.* C57Bl/6 mice may be involved as a compensatory mechanism to maintain vascular tone (Nayeem et al., 2013).

The conversion of arachidonic acid into epoxides takes place in the presence of CYP-epoxygenases, which have beneficial cardiovascular properties. Inhibition or deletion of CYP-epoxygenases including *Cyp2j5* may cause less EET-generation from arachidonic acid, leading to decreased vascular relaxation. Likewise, knocking out the *Cyp4a10* gene caused salt-sensitive hypertension with reduced EET excretion in the urine (Nakagawa et al., 2006). Some studies revealed that single nucleotide polymorphisms (SNPs) in the *CYP4A11* and *CYP2J2* genes were associated with human hypertension (Gainer et al., 2005; King et al., 2005). In addition, adenosine A_2A_ receptor (A_2A_ AR) knockout mice had lower expression of CYP-epoxygenases and higher expression of ω-hydroxylases with enhanced NECA (adenosine agonist) and CGS 21680 (A_2A_ AR-agonist)-induced vasoconstriction (aortic) compared to their respective wild-type mice (Nayeem et al., 2008; Pradhan et al., 2014; Pradhan et al., 2015). Further, Hanif et al. recently reported that A_2A_ AR^−/−^ mice had less plasma EETs compared to wild-type counterparts with reduced coronary reactive hyperemic response (Hanif et al., 2017a). An increase in blood pressure has been reported with enhanced renal vasoconstriction with angiotensin II in female *Cyp2j5*^−/−^ compared to female wild-type mice (Athirakul et al., 2008). To date, there are no studies addressing the vascular (aortic) response using acetylcholine and adenosine in male and female *Cyp2j5*^−/−^ *vs.* C57Bl/6 mice, though *Cyp2j5* enzyme is abundant in the kidneys as well as in mouse aorta where EETs get generated (Ma et al., 2004; Nayeem et al., 2008; Nayeem et al., 2013). Also, the effects of angiotensin-II in their vascular response in both *Cyp2j5*^−/−^ and wild type mice has not been investigated. Therefore, we hypothesized that *Cyp2j5*-gene deletion affects acetylcholine- and NECA-induced relaxation and their response is partially inhibited by Ang-II in mice.

## Materials and Methods

### Animals

Athirakul et al. (2008) described the generation of *Cyp2j5^−/−^* mice. *Cyp2j5^−/−^* and their respective C57Bl/6 mice were provided by Dr. Zeldin, National Institute of Environmental Health Sciences/National Institutes of Health (NIH). We used both male and female mice (14–18 week old) equally in our study.

### Materials

Phenylephrine, acetylcholine (ACh) (Sigma Chemicals, St. Louis, MO), and angiotensin II (Human), BACHEM (Bubendorf, Switzerland) dissolved in distilled water. *N*-(Methylsulfonyl)-2-(2-propynyloxy)-benzenehexanamide (MS-PPOH), dibromo-dodecenyl-methylsulfimide (DDMS) (Cayman Chemicals), 5'-N-ethylcarboxamidoadenosine (NECA), and CGS 21680 (Sigma Chemicals, St. Louis, MO) were dissolved in dimethyl sulfoxide (DMSO) (Nayeem et al., 2013).

### Muscle Bath Experiments

*Cyp2j5^−/−^* and C57Bl/6 mice were euthanized with pentobarbital sodium (100 mg/kg intraperitoneally). The aortas from *Cyp2j5^−/−^* and C57Bl/6 mice were removed after thoracotomy, the removed and cleaned aortas cut into four rings of 3–4 mm in length. The rings were hung between two wire hooks. The rings were suspended in organ baths of the tissue bath containing 10 ml of modified Krebs-Henseleit buffer in each bath. After the equilibration period (~60 min), aortic rings were contracted with KCl (50 mM) to assess the viability of the ring. Rings were then contracted with phenylephrine (PE; 10^−7^ M), and the ring tension was monitored continuously with a fixed range precision force transducer (125C; BIOPAC Systems) connected to amplifiers (Data Acquisition System 100B; BIOPAC Systems). Data were recorded using MP100 WSW, BIOPAC digital acquisition system and analyzed using Acknowledge 3.5.7 software (BIOPAC Systems). ACh (10^−12^–10^−5^ M) and NECA (adenosine, 10^−9^–10^−5^ M)-concentration dependent response experiments were conducted, as previously described with NECA, adenosine A_1_ receptor agonist (CCPA), and adenosine A_2A_ receptor agonist (CGS 21680) (Nayeem et al., 2008; Nayeem et al., 2009; Nayeem et al., 2010; Nayeem et al., 2011; Ponnoth et al., 2012a; Ponnoth et al., 2012b; Nayeem et al., 2013; Pradhan et al., 2014; Pradhan et al., 2015). The aortic rings were washed several times with Krebs-Henseleit solution and allowed to equilibrate for 30 min before the experimental protocol began. Results expressed as percentages (%) downward or upward on PE-induced precontracted rings.

Effects of N-(methylsulfonyl)-2-(2-propynyloxy)-benzenehexanamide (MS-PPOH) (CYP-epoxygenase inhibitor on ACh-induced colorectal carcinoma (CRC) in *Cyp2j5^−/−^* and C57Bl/6 mice. ACh-concentration dependent response curves (DDRCs) were obtained by cumulative addition of drugs in 1-log increments as described by us (Nayeem et al., 2008; Nayeem et al.,2009; Nayeem et al., 2010; Nayeem et al., 2011; Ponnoth et al., 2012a; Ponnoth et al., 2012b; Nayeem et al., 2013; Pradhan et al., 2014; Pradhan et al., 2015). A single DDRC was constructed for each ring in parallel in pairs of rings from either *Cyp2j5^−/−^* and C57Bl/6 mice in the same organ bath. ACh-DDRC was obtained with and without MS-PPOH (10^−5^ M), and MS-PPOH was added 30 min before the phenylephrine (PE) contraction and was present throughout the ACh DDRC. Similar as described earlier by us (Hanif et al., 2016b; Hanif et al., 2017b).

Effects of angiotensin-II (Ang-II, 1 µM) on ACh-induced DDRC in *Cyp2j5^−/−^* and C57Bl/6 mice. ACh-DDRC was obtained with and without Ang-II (1 µM), and Ang-II was added 30 min before the phenylephrine (PE) contraction and was present throughout the ACh-DDRC as described earlier by us (Ponnoth et al., 2012a; Yadav et al., 2015).

Effects of 20-HETE synthesis inhibitor, dibromo-dodecenyl-methylsulfimide (DDMS, 10^−5^ M) on adenosine (NECA) or CGS 21680-induced DDRC in *Cyp2j5^−/−^* and C57Bl/6 mice. NECA or CGS 21680-DDRC was obtained by cumulative addition of drugs in 1-log increments as described by us (Nayeem et al., 2008; Nayeem et al., 2009; Nayeem et al., 2010; Nayeem et al., 2011; Ponnoth et al., 2012a; Ponnoth et al., 2012b; Nayeem et al., 2013; Pradhan et al., 2014; Pradhan et al., 2015). A single DDRC was constructed for each ring in parallel in pairs of rings from either *Cyp2j5^−/−^* and C57Bl/6 mice in the same organ bath. NECA or CGS 21680-DDRC was obtained with and without DDMS (10^−5^ M), and DDMS was added 30 min before the PE contraction and was present throughout the NECA or CGS 21680-DDRC.

Effects of angiotensin-II (Ang-II, 10^−6^ M) on top of DDMS in NECA or CGS 21680-induced DDRC in *Cyp2j5^−/−^* and C57Bl/6 mice. NECA or CGS 21680-induced DDRC was obtained with and without Ang-II (10^−6^ M) and DDMS (10^−5^ M), and Ang-II ± DDMS was added 30 min before the PE contraction and was present throughout the NECA or CGS 21680-induced DDRC. Similar as described earlier by us (Nayeem et al., 2009; Nayeem et al., 2010; Nayeem et al., 2011; Ponnoth et al., 2012a; Yadav et al., 2015).

### Statistical Analysis

The data reported as means ± SE. One-way ANOVA was used to compare difference among groups, and two-way ANOVA was used for repeated measures, followed by Tukey *post hoc* test to compare the ACh/NECA/CGS 21680-induced vascular responses of non-treated *vs.* inhibitor/antagonist (MS-PPOH, DDMS, and Ang-II)-treated *Cyp2j5^−/−^* and C57Bl/6 mice. Differences (*Cyp2j5^−/−^ vs*. C57Bl/6), non-treated *vs.* (MS-PPOH, DDMS, and Ang-II)-treated *Cyp2j5^−/−^* and C57Bl/6 mice were considered significant when *P <* 0.05. All the statistical analyses performed using GraphPad Prism statistical package.

## Results

**Figure 1:**
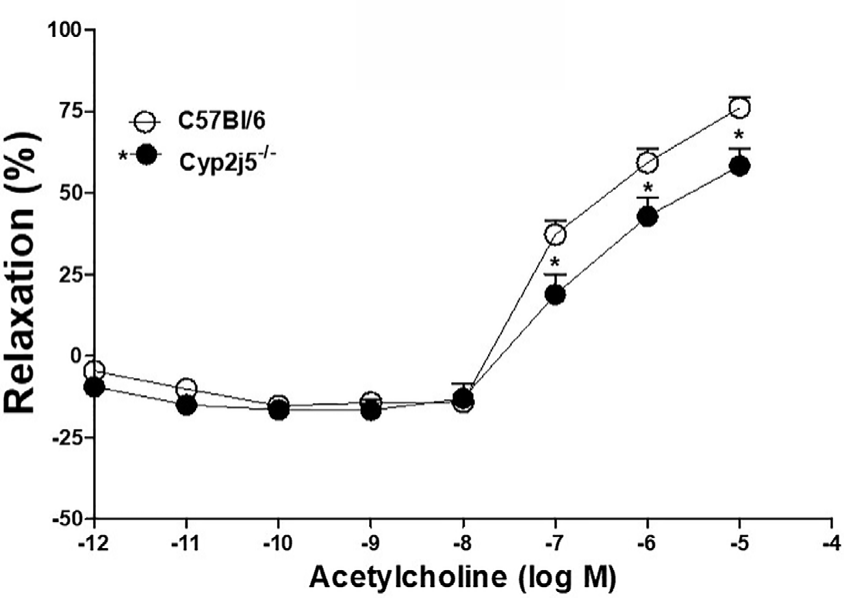
Acetylcholine (ACh)-induced concentration dependent vascular response in aortic rings of C57Bl/6 and *Cyp2j5^−/−^* mice. *P < 0.05 between C57Bl/6 *vs.* *Cyp2j5^−/−^* mice, there is a significant difference found in (ACh)-induced concentration (10^−7^M–10^−5^M) dependent vascular response between C57Bl/6 and *Cyp2j5^−/−^* mice (*P < 0.05). Values expressed as means ± SE. *n* = eight per group.

Concentration dependent response curve (DDRC) for ACh-induced relaxation in *Cyp2j5^−/−^* and C57Bl/6 mice: ACh caused a concentration (10^−7^–10^−5^ M)-dependent relaxation in both C57Bl/6 and *Cyp2j5^−/^*^−^ mice. The response was significantly different (ACh-10^−7^–10^−6^ M, *P <* 0.05) between C57Bl/6 *vs. Cyp2j5^−/−^* mice (**Figure 1**).

**Figure 2:**
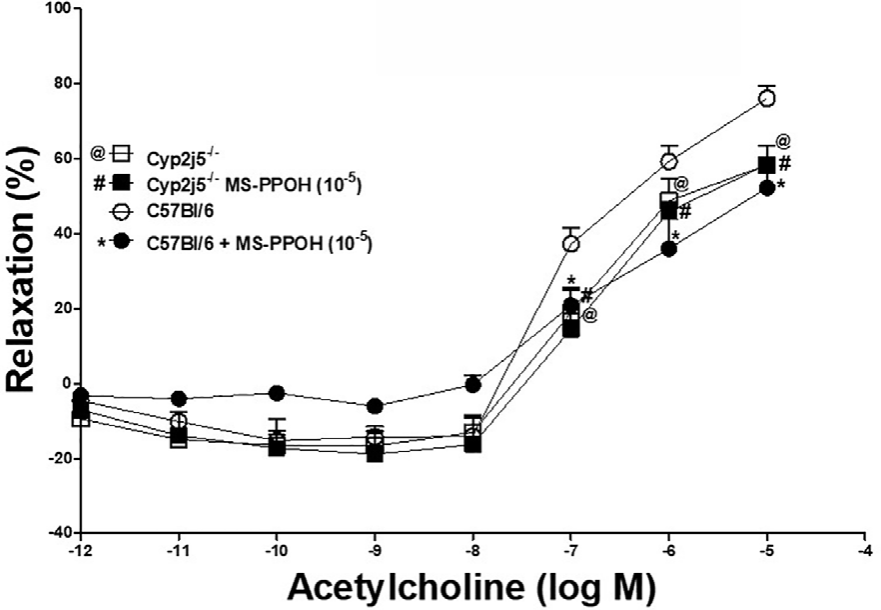
Effect of MS-PPOH (10^−5^ M) on ACh-induced concentration dependent vascular response in aortic rings of C57Bl/6 and Cyp2j5^−/−^ mice. *P < 0.05 between non-treated C57Bl/6 *vs.* MS-PPOH treated C57Bl/6 mice, ^@^*P* < 0.05 between non-treated C57Bl/6 *vs.* non-treated *Cyp2j5*^−/−^ mice, ^#^*P* < 0.05 between non-treated C57Bl/6 *vs.* MS-PPOH-treated *Cyp2j5*^−/−^ mice. ^#@^*P* > 0.05 between non-treated *Cyp2j5^−/−^* and MS-PPOH treated *Cyp2j5^−/−^* mice and between MS-PPOH treated *Cyp2j5^−/−^ vs*. MS-PPOH treated *C57Bl/6* mice (^#*^P > 0.05). Values expressed as means ± SE. *n* = eight per group.

DDRC for ACh with or without MS-PPOH (CYP-epoxygenases inhibitor, 10^−5^ M) treated C57Bl/6 and *Cyp2j5^−/−^* mice: at 10^−5^ M ACh, MS-PPOH reduced the ACh-induced concentration-dependent relaxation in C57Bl/6 (**Figure 2**). There was no change in treated *Cyp2j5^−/−^* mice *vs.* non-treated (*P* > 0.05, **Figure 2**). However, there was a significant change between C57Bl/6 *vs. Cyp2j5^−/−^* mice (at 10^−5^ M ACh, 76.1 ± 3.3 *vs.* 58.3 ± 5.2, *P* < 0.05, **Figure 2**).

**Figure 3:**
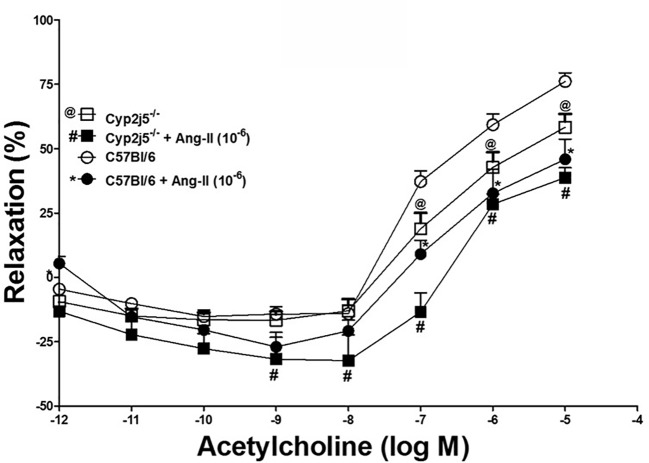
Effect of angiotensin II (Ang-II) (10^−6^ M) on acetylcholine (Ach)-induced concentration dependent vascular response in aortic rings of C57Bl/6 and Cyp2j5^−/−^ mice. *P < 0.05 between non-treated C57Bl/6 *vs.* Ang-II treated C57Bl/6 mice, ^@^*P* < 0.05 between non-treated C57Bl/6 *vs.* non-treated *Cyp2j5*^−/−^ mice, ^#^*P* < 0.05 between non-treated C57Bl/6 *vs.* Ang-II-treated *Cyp2j5*^−/−^ mice. ^#@^*P* < 0.05 between non-treated *Cyp2j5^−/−^* and Ang-II treated *Cyp2j5^−/−^* mice and between Ang-II treated *Cyp2j5^−/−^ vs*. Ang-II treated *C57Bl/6* mice (^#*^P > 0.05). Values expressed as means ± SE. *n* = eight per group.

DDRC for ACh and the effects of Ang-II (10^−6^ M) in *Cyp2j5^−/−^* and C57Bl/6 mice: ACh produced a concentration-dependent relaxation in both *Cyp2j5^−/−^* and C57Bl/6 mice. Ang-II (10^−6^ M) attenuated ACh-induced concentration-dependent relaxation in both C57Bl/6 and *Cyp2j5-/-*treated *vs.* non-treated (*P <* 0.05, **Figure 3**) mice. Interestingly, Ang-II treatment attenuated more ACh-induced concentration-dependent relaxation in *Cyp2j5^−/−^ vs*. Ang-II treated C57Bl/6 (*P <* 0.05, **Figure 3**). In addition, the effect of Ang-II was significantly different at the low concentrations of ACh 10^−9^–10^−8^ M in *Cyp2j5^−/−^ vs*. non-treated *Cyp2j5^−/−^* mice (*P* < 0.05, **Figure 3**).

**Figure 4:**
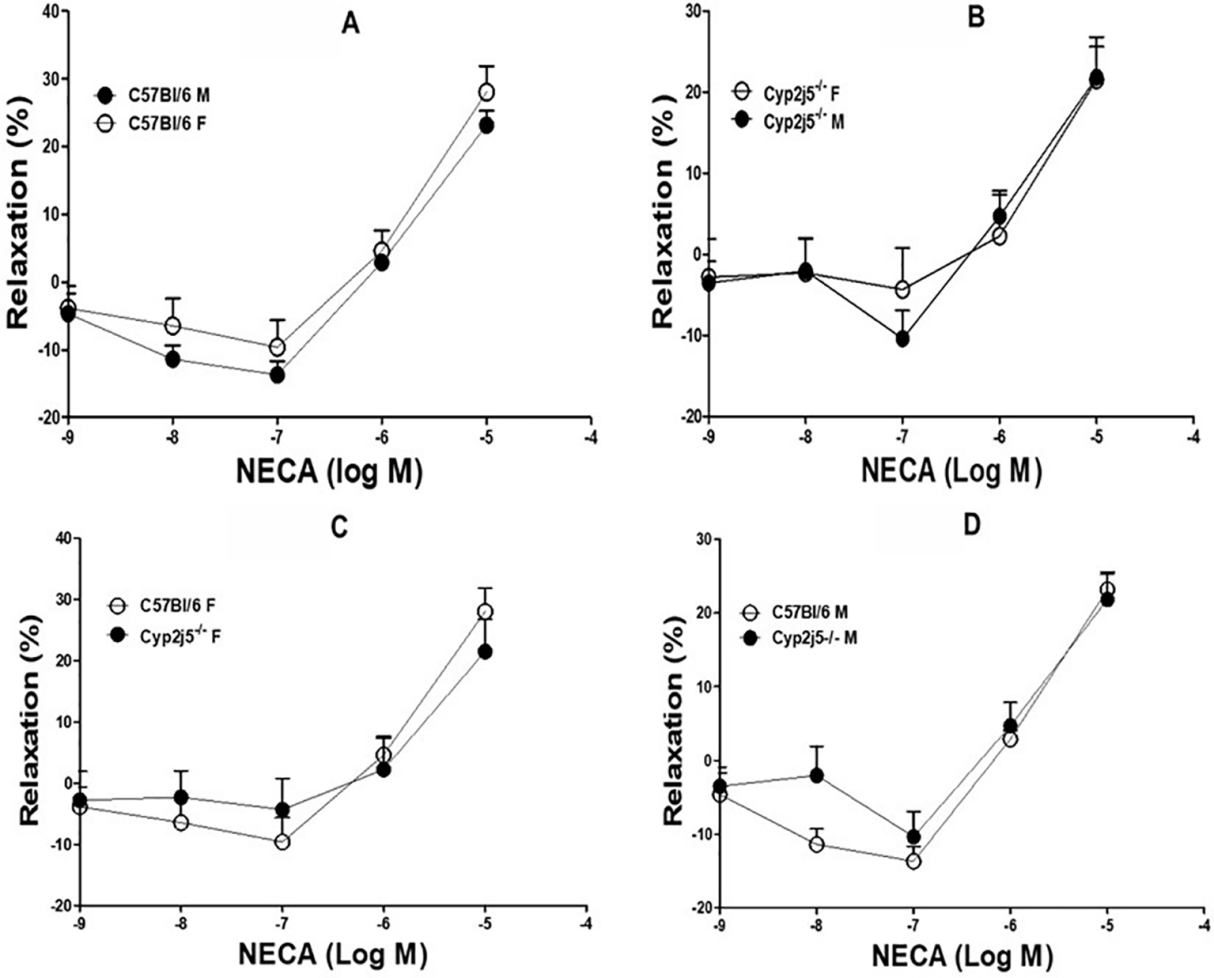
5'-N-Ethylcarboxamidoadenosine (NECA)-induced concentration dependent vascular response in aortic rings of C57Bl/6 and *Cyp2j5^−/−^* mice. *P* > 0.05 between C57Bl/6 (M) *vs.* C57Bl/6 (F) mice **(A)**, *P* > 0.05 between *Cyp2j5*^−/−^ (M) *vs.* *Cyp2j5*^−/−^ (F) mice **(B)**, *P* > 0.05 between *Cyp2j5*^−/−^ (F) *vs.* C57Bl/6 (F) mice **(C)** and *P* > 0.05 between *Cyp2j5*^−/−^ (M) *vs.* C57Bl/6 (M) mice **(D)**. Values expressed as means ± SE. *n* = eight per group.

DDRC for adenosine (NECA)-induced relaxation in *Cyp2j5^−/−^* and C57Bl/6 mice: NECA caused a concentration (10^−7^–10^−5^ M)-dependent relaxation, but the response was not different between male and female C57Bl/6 mice (NECA-10^−7^–10^−6^ M, **Figure 4A**, *P >* 0.05) or *Cyp2j5^−/^*^−^ mice (NECA-10^−7^–10^−6^ M, **Figures 4B–D**, *P >* 0.05).

**Figure 5:**
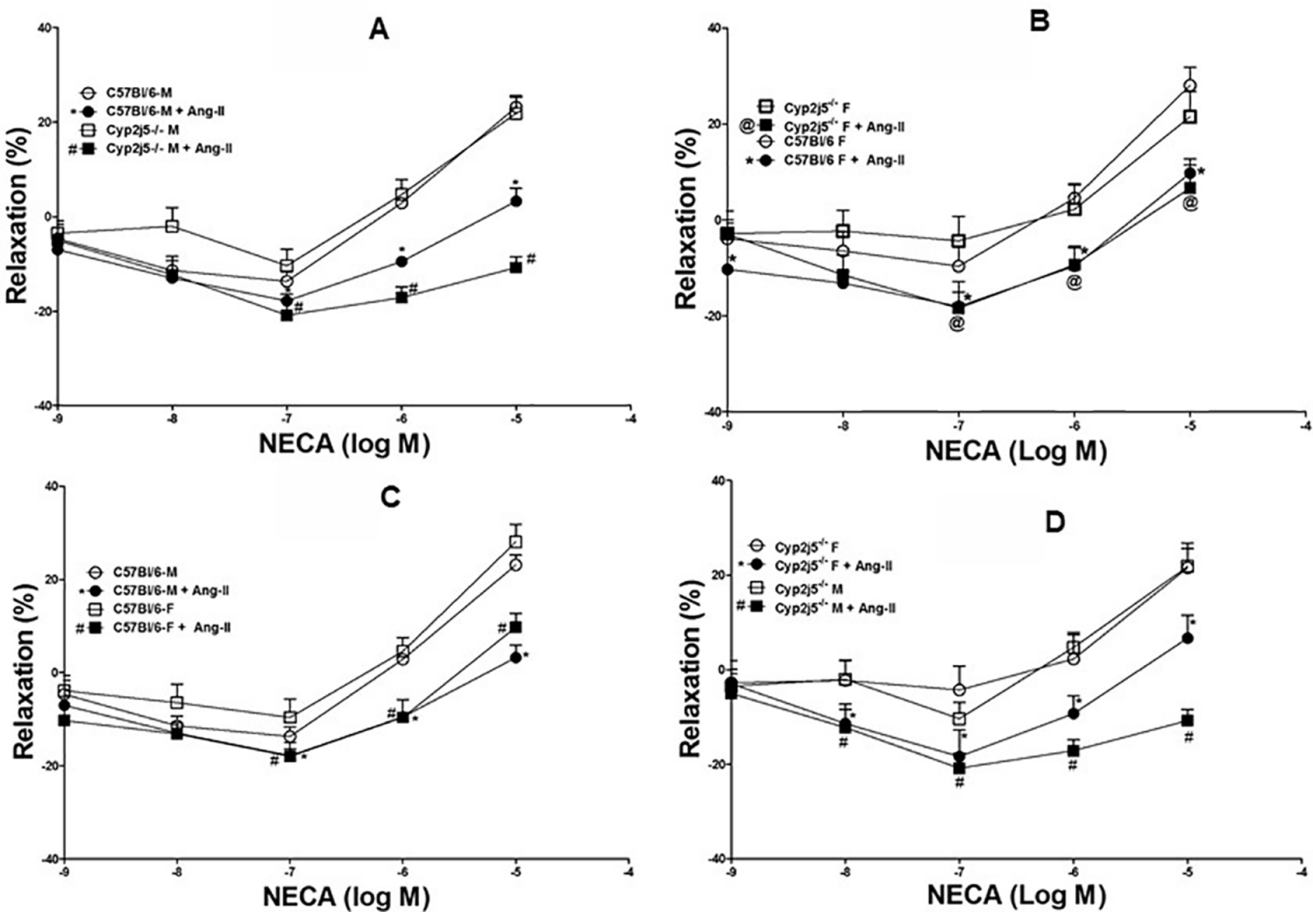
**(A)** Effect of angiotensin II (Ang-II) (10^−6^ M) on 5'-N-ethylcarboxamidoadenosine (NECA)-induced concentration dependent vascular response in aortic rings of C57Bl/6 (M) and Cyp2j5^−/−^ (M) mice. *P < 0.05 between non-treated C57Bl/6 (M) *vs.* Ang-II treated C57Bl/6 (M) mice, *P* > 0.05 between non-treated C57Bl/6 (M) *vs.* non-treated *Cyp2j5*^−/−^ (M) mice, ^#^*P* < 0.05 between non-treated C57Bl/6 (M) *vs.* Ang-II-treated *Cyp2j5*^−/−^ (M) mice. ^#^*P* < 0.05 between non-treated *Cyp2j5^−/−^* (M) and Ang-II treated *Cyp2j5^−/−^* (M) mice and between Ang-II treated *Cyp2j5^−/−^* (M) *vs.* Ang-II treated *C57Bl/6* (M) mice (^#*^P < 0.05). Values expressed as means ± SE. *n* = eight per group (5A). **(B)** Ang-II effect was tested on NECA-induced response in C57Bl/6 (F) and Cyp2j5^−/−^ (F) mouse aortas. *P < 0.05 between non-treated C57Bl/6 (F) *vs.* Ang-II treated C57Bl/6 (F) mice, *P* > 0.05 between non-treated C57Bl/6 (F) *vs.* non-treated *Cyp2j5*^−/−^ (F) mice, ^@^*P* < 0.05 between non-treated C57Bl/6 (F) *vs.* Ang-II-treated *Cyp2j5*^−/−^ (F) mice. ^@^*P* < 0.05 between non-treated *Cyp2j5^−/−^* (F) and Ang-II treated *Cyp2j5^−/−^* (F) mice and between Ang-II treated *Cyp2j5^−/−^* (F) *vs.* Ang-II treated *C57Bl/6* (F) mice (^@*^P > 0.05). Values expressed as means ± SE. *n* = eight per group (5B). **(C)** NECA-induced concentration dependent vascular response was tested with Ang-II (10^−6^ M) in aortic rings of C57Bl/6 (M) and C57Bl/6 (F) mice. *P < 0.05 between non-treated C57Bl/6 (M) *vs.* Ang-II treated C57Bl/6 (M) mice, *P* > 0.05 between non-treated C57Bl/6 (M) *vs.* non-treated C57Bl/6 (F) mice, ^#^*P* < 0.05 between non-treated C57Bl/6 (F) *vs.* Ang-II-treated C57Bl/6 (F) mice. ^@^*P* < 0.05 between non-treated *C57Bl/6* (F) and Ang-II treated *C57Bl/6* (F) mice and between Ang-II treated *C57Bl/6* (F) *vs.* Ang-II treated *C57Bl/6* (M) mice (^#*^P > 0.05). Values expressed as means ± SE. *n* = eight per group **(C)**. **(D)** Effects of Ang-II on NECA-induced concentration dependent vascular response in aortic rings of *Cy2j5^−/−^* (M) and *Cy2j5^−/−^* (F) mice. #*P* < 0.05 between non-treated *Cy2j5^−/−^* (M) *vs.* Ang-II treated *Cy2j5^−/−^* (M) mice, *P* > 0.05 between non-treated *Cy2j5^−/−^* (M) *vs.* non-treated *Cy2j5^−/−^* (F) mice, *P < 0.05 between non-treated *Cy2j5^−/−^* (F) *vs.* Ang-II-treated *Cy2j5^−/−^* (F) mice. ^#^*P* < 0.05 between non-treated *Cy2j5^−/−^* (M) and Ang-II treated *Cy2j5^−/−^* (F) mice, and between Ang-II treated *Cy2j5^−/−^* (F) *vs.* Ang-II treated *Cy2j5^−/−^* (M) mice (^#^*P < 0.05). Values expressed as means ± SE. *n* = eight per group (5D).

DDRC for NECA and the effect of Ang-II (10^−6^ M) on male *Cyp2j5^−/−^ vs*. male C57Bl/6 mice: NECA produced a concentration-dependent relaxation in both male *Cyp2j5^−/^*^−^ and male C57Bl/6 mice. Ang-II (10^−6^ M) attenuates NECA-induced concentration-dependent relaxation in both male C57Bl/6 (at NECA 10^−7^–10^−5^ M; −17.8 ± 1.4, −*9.5 ± 1.1, *3.2 ± 2.7 *vs.* non-treated −13.7 ± 2.0, 2.9 ± 1.1, 23.1 ± 2.1, *P < 0.05, **Figure 5A**) and male *Cyp2j5^−/−^* (at NECA 10^−7^–10^−5^ M; −^#^20.85 ± 3.2, −^#^17.1± 2.3, −^#^10.8 ± 2.3 *vs.* non-treated −10.4 ± 3.5, 4.7 ± 3.1, 21.8 ± 3.8, ^#^*P* < 0.05, **Figure 5A**) mice. Interestingly, Ang-II treatment attenuates NECA-induced concentration-dependent relaxation in *Cyp2j5^−/−^ vs*. Ang-II treated C57Bl/6 mice (*P <*0.05, **Figure 5A**). However, no significant difference was observed between male *Cyp2j5^−/−^ vs*. male C57Bl/6 mice (NECA-10^−7^–10^−6^ M, **Figure 5A**, *P >*0.05).

DDRC for NECA and the effects of Ang-II (10^−6^ M) in female *Cyp2j5^−/−^ vs*. female C57Bl/6 mice: NECA produced a concentration-dependent relaxation in both female *Cyp2j5^−/−^* and female C57Bl/6 mice. Ang-II (10^−6^ M) attenuates NECA-induced concentration-dependent relaxation in both female C57Bl/6 (at NECA 10^−7^–10^−5^ M; −*18.00 ± 3.02, −*9.6 ± 3.8, *9.7 ± 3.0 *vs.* non-treated −9.6 ± 4.0, 4.6 ± 3.0, 28.0 ± 3.8, *P < 0.05, **Figure 5B**) and female *Cyp2j5^−/−^* (at NECA 10^−7^–10^−5^ M; −^@^18.4 ± 5.6, −^@^9.3± 3.8, ^@^6.6 ± 4.8 *vs.* non-treated −4.3 ± 5.1, 2.2 ± 5.1, 21.5 ± 5.3, ^@^*P* < 0.05, **Figure 5B**) mice. Ang-II treatment attenuates NECA-induced concentration-dependent relaxation equally in both in female *Cyp2j5^−/−^* and female C57Bl/6 mice *vs.* non-treated female *Cyp2j5^−/−^* and female C57Bl/6 mice (*P <* 0.05, **Figure 5B**). However, no significant difference was observed between female *Cyp2j5^−/−^ vs*. female C57Bl/6 mice (NECA-10^−7^–10^−6^ M, **Figure 5B**, *P >* 0.05).

DDRC for NECA and the effects of Ang-II (10^−6^ M) in male C57Bl/6 *vs.* female C57Bl/6 mice: NECA produced a concentration-dependent relaxation in both male C57Bl/6 and female C57Bl/6 mice. Ang-II (10^−6^ M) attenuates NECA-induced concentration-dependent relaxation in both female C57Bl/6 (at NECA 10^−7^–10^−5^ M; −*18.0 ± 3.0, −*9.6 ± 3.8, *9.7 ± 3.0 *vs.* non-treated −9.6 ± 4.0, 4.6 ± 3.0, 28.0 ± 3.8, *P < 0.05, **Figure 5C**) and male C57Bl/6 (at NECA 10^−7^–10^−5^ M; −^#^17.8 ± 1.4, −^#^9.5± 1.1, ^#^3.2 ± 2.7 *vs.* non-treated −13.7 ± 2.0, 2.9 ± 1.1, 23.1 ± 2.1, ^#^*P* < 0.05, **Figure 5C**) mice. Ang-II treatment attenuates NECA-induced concentration-dependent relaxation equally in both in female C57Bl/6 and male C57Bl/6 mice *vs.* non-treated female and male C57Bl/6 mice (*P <* 0.05, **Figure 5C**). However, no significant difference was observed between female C57Bl/6 *vs.* male C57Bl/6 mice (NECA-10^−7^–10^−5^ M, **Figure 5C**, *P >* 0.05).

DDRC for NECA and the effects of Ang-II (10^−6^ M) in male *Cyp2j5^−/−^ vs*. female *Cyp2j5^−/−^* mice: NECA produced a concentration-dependent relaxation in both male *Cyp2j5^−/−^* and female *Cyp2j5^−/−^* mice. Ang-II (10^−6^ M) attenuates NECA-induced concentration-dependent relaxation in both female *Cyp2j5^−/−^* (at NECA 10^−7^–10^−5^ M; −*18.4 ± 5.6, −*9.3 ± 3.8, *6.6 ± 4.8 *vs.* non-treated −4.3 ± 5.1, 2.2 ± 5.1, 21.5 ± 5.3, *P < 0.05, **Figure 5D**) and male *Cyp2j5^−/−^* (at NECA 10^−7^–10^−5^ M; −^#^20.8 ± 3.2, −^#^17.1± 2.3, −^#^10.8 ± 2.3 *vs.* non-treated −10.4 ± 3.5, 4.7 ± 3.1, 21.8 ± 3.8, ^#^*P* < 0.05, **Figure 5D**) mice. Interestingly, Ang-II treatment attenuates more NECA-induced concentration-dependent relaxation in male *Cyp2j5^−/−^* mice *vs.* female *Cyp2j5^−/−^* mice (*P <*0.05, **Figure 5D**). However, no significant difference was observed between female *Cyp2j5^−/−^ vs*. male *Cyp2j5^−/−^* mice (NECA-10^−7^–10^−6^ M, **Figure 5D**, *P >* 0.05).

DDRC for CGS 21680 (A_2A_ AR-agonist) in DDMS (10^−5^ M, 20-HETE synthesis inhibitor) + Ang-II (10^−6^ M) in male *Cyp2j5^−/−^ vs*. non-treated male *Cyp2j5^−/−^* mice: CGS 21680 produced an enhanced concentration-dependent relaxation in male DDMS-treated *Cyp2j5^−/−^* mice (at CGS 21680 10^−7^–10^−5^ M; 9.7 ± 2.5, 21.5 ± 2.8, 30.5 ± 2.7) *vs.* non-treated male *Cyp2j5^−/−^* mice (at CGS 21680 10^−7^–10^−5^M; −^@^1.6 ± 1.1, ^@^12.7 ± 1.1, ^@^20.5 ± 1.8, ^@^*P* < 0.05). In addition, no significant change was observed between DDMS + Ang-II treated male *Cyp2j5^−/−^* mice (at CGS 21680 10^−7^–10^−5^M; −6.4 ± 1.7, 9.4 ± 1.7, 15.8 ± 1.9) *vs.* non-treated male *Cyp2j5^−/−^* mice (at CGS 21680 10^−7^–10^−5^M; −1.6 ± 1.1, 12.7 ± 1.1, 20.5 ± 1.8, *P* > 0.05). However, a significant difference was observed between DDMS-treated male *Cyp2j5^−/−^* mice (at CGS 21680 10^−7^–10^−5^M; 9.7 ± 2.5, 21.5 ± 2.8, 30.5 ± 2.7) *vs.* DDMS + Ang-II treated male *Cyp2j5^−/−^* mice (at CGS 21680 10^−7^–10^−5^M; −^*^6.4 ± 1.7, ^*^9.4 ± 1.7, ^*^15.8 ± 1.9, ^*^P < 0.05). Similar trends were observed earlier in DDMS treated *vs.* non-treated C57Bl/6 mice (Nayeem et al., 2009; Nayeem et al., 2010; Nayeem et al., 2011; Ponnoth et al., 2012a; Yadav et al., 2015).

DDRC for CGS 21680 in DDMS (10^−5^ M) + Ang-II (10^−6^ M) in female *Cyp2j5^−/−^ vs*. non-treated female *Cyp2j5^−/−^* mice: CGS 21680 produced an enhanced concentration-dependent relaxation in female DDMS-treated *Cyp2j5^−/−^* mice (at CGS 21680 10^−7^–10^−5^M; 7.9 ± 2.1, 17.2 ± 2.3, 25.2 ± 2.4) *vs.* non-treated female *Cyp2j5^−/−^* mice (at CGS 21680 10^−7^–10^−5^M; −4.6 ± 1.1, 5.7 ± 1.1, 15.5 ± 1.8, ^@^*P* < 0.05). In addition, no significant change was observed between DDMS + Ang-II treated female *Cyp2j5^−/−^* mice (at CGS 21680 10^−7^–10^−5^M; −15.8 ± 2.6, 2.3 ± 1.2, 9.2 ± 1.5) *vs.* non-treated female *Cyp2j5^−/−^* mice except at 10^−7^ M (at CGS 21680 10^−7^–10^−5^ M; −*4.6 ± 1.1, 5.7 ± 1.1, 15.5 ± 1.8, *P < 0.05). Whereas, a significant difference was observed between female DDMS-treated *Cyp2j5^−/−^* mice (at CGS 21680 10^−7^–10^−5^ M; 7.9 ± 2.1, 17.2 ± 2.3, 25.2 ± 2.4) *vs.* DDMS + Ang-II treated female *Cyp2j5^−/−^* mice (at CGS 21680 10^−7^–10^−5^ M; −*15.8 ± 2.6, *2.3 ± 1.2, *9.2 ± 1.5, ^*^P < 0.05). Similar trends were observed earlier in DDMS treated *vs.* non-treated C57Bl/6 mice (Nayeem et al., 2009; Nayeem et al., 2010; Nayeem et al., 2011; Ponnoth et al., 2012a; Yadav et al., 2015).

## Discussion

This is the first study to investigate the ACh-, adenosine (NECA)-, and A_2A_ AR agonist (CGS 21680)-induced concentration-dependent vascular (aortic) response and their interactions among CYP-epoxygenases, ω-hydroxlases and Ang-II in male *vs.* female *Cyp2j5^−/−^* mice and *Cyp2j5^−/−^ vs*. C57Bl/6 mice. We report novel findings regarding the vascular (aortic) response between *Cyp2j5^−/−^ vs*. C57Bl/6 mice and their interactions among CYP-epoxygenase, ω-hydroxlase and Ang-II. (1) Disruption of *Cyp2j5* gene in mice (*Cyp2j5^−/−^*) reduced ACh-induced concentration-dependent relaxation compared to C57Bl/6 mice. Thus, it is clear that *Cyp2j5* has its own role in ACh-induced vascular response. Loss of *Cyp2j5* showed reduced ACh-induced relaxation, and *Cyp2j5* protein is also detected in kidneys/mouse aorta/visceral adipose tissues, and is involved in the conversion of AAs into EETs (Ma et al., 2004; Burgess et al., 2012; Nayeem et al., 2013). (2) CYP-epoxygenase inhibitor (MS-PPOH) partially attenuated ACh-induced concentration dependent relaxation in C57Bl/6, but not in *Cyp2j5^−/−^* mice. Thus, *Cyp2j5* enzyme activity is involved in ACh-induced concentration dependent relaxation in C57Bl/6 mice. (3) Ang-II attenuates ACh-induced concentration dependent relaxation in *Cyp2j5^−/−^* compared to C57Bl/6 mice. Therefore, we conclude that *Cyp2j5* enzyme is involved in ACh-induced concentration dependent vascular response in Ang-II–treated mice. (4) There was no difference found between the presence and absence of *Cyp2j5* in mice related to adenosine (NECA)-induced vascular response. (5) Ang-II treatment significantly attenuated NECA-induced concentration dependent relaxation in *Cyp2j5^−/−^* (M) and C57Bl/6 (M) mice, and the attenuation of NECA-induced concentration dependent relaxation due to Ang-II infused *Cyp2j5^−/−^* (M) is much higher than C57Bl/6 (M) mice. Therefore, presence of *Cyp2j5* in C57Bl/6 (M) mice plays an important role in resisting the reduction of NECA-induced concentration dependent relaxation due to Ang-II in male mice. (6) Ang-II treatment was able to significantly attenuate NECA-induced concentration dependent relaxation in both *Cyp2j5^−/−^* (F) and C57Bl/6 (F) mice, and a similar effect was observed in *Cyp2j5^−/−^* (M) *vs.* C57Bl/6 (M) mice after Ang-II treatment *vs.* non-treated. (7) However, the attenuation of NECA-induced concentration dependent relaxation due to Ang-II treated *Cyp2j5^−/−^* (M) is much higher than *Cyp2j5^−/−^* (F) mice. Therefore, the *Cyp2j5^−/−^* (M) may be more sensitive to Ang-II in NECA-induced response compared to *Cyp2j5^−/−^* (F) mice. (8) ω-hydroyxlase inhibitor (DDMS) enhances A_2A_AR (CGS 21680)-induced concentration dependent relaxation in both male and female *Cyp2j5^−/−^* mice compared non-treated male and female *Cyp2j5^−/−^* mice, and DDMS + Ang-II treated male/female *Cyp2j5^−/−^* mice are not different from the non-treated male/female *Cyp2j5^−/−^* mice in regards to their CGS 21680-induced concentration dependent vascular response. Thus, ω-hydroyxlases are involved in the attenuation of CGS 21680-induced concentration dependent vascular relaxation while Ang-II infused in both male and female *Cyp2j5^−/−^* mice. According to our data, *Cyp2j5* plays an important role in the in ACh-, NECA-, and CGS 21680-induced concentration dependent regulation of vascular response.

CYP450-epoxygenase (CYP2J2) polymorphisms have been reported in different populations related to its role in cardiovascular function, including hypertension (Lee et al., 2007; Feng et al., 2008; Fava et al., 2010; Jie et al., 2010; Wang et al., 2010; Zordoky and El-Kadi, 2010; Xu et al., 2011). The *Cyp2j5*-epoxygenase in mouse (chromosome-4) appears to be as important as *CYP2J2*-epoxygenase in human (chromosome-1) and CYP2J4 in rat (chromosome-5) (Olona et al., 2018). Variations in soluble epoxide hydrolase and ω-hydroxylase genes in human population also alter the risk of coronary heart disease, ischemic stroke, restenosis, diabetes heart, heart failure, ischemic stroke in Caucasians, Chinese, and in the African Americans with hypertension (Lee et al., 2006; Burdon et al., 2008; Monti et al., 2008; Kullmann et al., 2009; Fava et al., 2010; Zordoky and El-Kadi, 2010).

Role of *Cyp2j5* in ACh-induced vascular response: ACh-induced concentration dependent aortic relaxation in *Cyp2j5^−/−^* was significantly different compared to C57Bl/6 mice (**Figure 1**). ACh data between *Cyp2j5^−/−^* and C57Bl/6 mice suggest that lack of *Cyp2j5*-epoxygenase (less EETs generation) contributes less in ACh-induced concentration dependent aortic relaxation in *Cyp2j5^−/−^* compared to C57Bl/6 mice, and ACh-induced relaxation is almost completely dependent on NO. As Hercule, et al. indicated, CYP450-eicosanoids activates endothelial nitric oxide (NO) synthase and NO release in *Ephx2^−/−^* (more EETs) and wild-type (WT) mouse mesenteric arteries (Hercule et al., 2009). In addition, EETs have been demonstrated to increase NO release; in bovine aortic endothelial cells (cultured) EETs can induce NO release, which may modulate vascular tone (Wang et al., 2003; Hercule et al., 2009). Therefore, according to the current study, we believe *Cyp2j5^−/−^* mice, which lack *Cyp2j5*, may have reduced EET formation and subsequent ACh-induced and NO-dependent aortic relaxation compared to C57Bl/6 mice. Because, we found overexpression of *Cyp2j5* protein and up-regulation of EET oxylipins (isolated heart perfusate) in *Ephx2^−/−^* compared to C57Bl/6 mice with enhanced aortic and coronary reactive hyperemic response (Nayeem et al., 2013; Hanif et al., 2016a), and endothelial vascular overexpression of human CYP2J2 had up-regulation of EET oxylipins (isolated heart perfusate) in CYP2J2 (Tie2-CYP2J2 Tr) compared to C57Bl/6 mice with enhanced coronary reactive hyperemic response (Hanif et al., 2017b).

Role of CYP-epoxygenases in ACh-induced relaxation: MS-PPOH (CYP-epoxygenases inhibitor) was able to partially block ACh-induced concentration dependent aortic relaxation in C57Bl/6 mice, but not in *Cyp2j5^−/−^* compared to non-treated mice (**Figure 2**). There is no difference was found between MS-PPOH treated *Cyp2j5^−/−^ vs*. non-treated *Cyp2j5^−/−^* mice. This suggests that *Cyp2j5* is a main contributor in the formation of epoxides from AA in mouse aorta, as demonstrated previously that mouse kidneys have higher *Cyp2j5* expression in male compared to female mice after puberty (Ma et al., 2004), and Northern analysis also revealed that *Cyp2j5* transcripts were more abundant in adult male *versus* adult female kidneys (Ma et al., 2004). Burgess *et al*., showed that CYP2J5 is responsible for production of primarily 14,15 and 11,12 EETs in visceral adipose tissue (Burgess et al., 2012), and the overexpression of *Cyp2j5* and *Cyp4a* proteins were observed in *Ephx2*^−/−^ *vs.* C57Bl/6 mice with enhanced adenosine (NECA) and CGS 21680 (A_2A_ AR)-induced aortic relaxation (Nayeem et al., 2013). Relative loss of ACh-induced concentration dependent aortic relaxation in *Cyp2j5*^−/−^ *vs.* C57Bl/6 mice and no change between MS-PPOH treated *Cyp2j5^−/−^ vs*. non-treated *Cyp2j5^−/−^* mice indicates a central role of CYP-epoxygenases like *Cyp2j5* in ACh-induced concentration dependent aortic relaxation. This is similar to that reported in mouse mesenteric arteries (Hercule et al., 2009).

Effect of Ang-II in ACh-induced relaxation: Ang-II was able to partially block ACh-induced concentration dependent aortic relaxation in both *Cyp2j5^−/−^* and C57Bl/6 mice compared to non-treated mice (**Figure 3**), and a significant difference was found between the Ang-II-treated C57Bl/6 *vs.* non-treated C57Bl/6 mice and Ang-II-treated *Cyp2j5^−/−^ vs*. non-treated *Cyp2j5^−/−^* mice (**Figure 3**). Attenuation of ACh-induced concentration dependent aortic relaxation was greater in Ang-II-treated *Cyp2j5^−/−^ vs*. non-treated C57Bl/6 mice compared to Ang-II-treated C57Bl/6 *vs.* non-treated C57Bl/6 mice. This shows that the lack of *Cyp2j5* gene in *Cyp2j5^−/−^* mice has less advantage over C57Bl/6 to block the action of Ang-II in ACh-induced concentration dependent aortic relaxation.

Deletion of *Cyp2j5* (*Cyp2j5^−/−^*) or inhibition (through MS-PPOH) of CYP-epoxygenases activity causes less EET generation from arachidonic acid (AA) metabolism and leads to a decrease in ACh-concentration dependent relaxation. Ang-II blocked ACh-induced concentration dependent relaxation in both *Cyp2j5^−/−^* and C57Bl/6 mice. However, in the absence of *Cyp2j5*, Ang-II blocking action is significantly more than the blocking action in C57Bl/6 mice. Therefore, we conclude that inhibition or deletion of CYP-epoxygenases in mice may enhance the toxic action of Ang-II in ACh-induced concentration dependent relaxation in *Cyp2j5^−/−^* compared to C57Bl/6 mice. Also, deletion or inhibition of CYP-epoxygenases activity causes less EETs to form and involved in vascular contraction, reduced coronary reactive hyperemic response with the association of vascular inflammation (Nayeem et al., 2008; Nayeem et al., 2009; Nayeem et al., 2010; Nayeem et al., 2011; Ponnoth et al., 2012b; Hanif et al., 2016b; Hanif et al., 2017b).

Role of *Cyp2j5* in NECA-induced vascular response and effect of Ang-II in NECA-induced vascular response: there was no difference between the presence or absence of *Cyp2j5* in both male *vs.* female or male *vs.* female C57Bl/6 mice regarding NECA-induced concentration dependent relaxation (**Figures 4A–D**), which is not similar to ACh-induced concentration dependent vascular response where *Cyp2j5^−/−^* had lesser relaxation compared C57Bl/6 mice (**Figure 1**). But, Ang-II treatment was able to block NECA-induced concentration dependent aortic relaxation in both male *Cyp2j5^−/−^* and male C57Bl/6 mice compared to non-treated male mice (**Figure 5A**), and a large difference was found between the Ang-II-treated male C57Bl/6 *vs.* non-treated male C57Bl/6 mice and a significant difference was observed between Ang-II-treated male *Cyp2j5^−/−^ vs*. non-treated male *Cyp2j5^−/−^* mice (**Figure 5A**). Attenuation of NECA-induced concentration dependent aortic relaxation was greater in Ang-II-treated male *Cyp2j5^−/−^ vs*. Ang-II-treated male C57Bl/6 mice (**Figure 5A**). In addition, there was significant difference observed between Ang-II-treated male *Cyp2j5^−/−^ vs*. Ang-II-treated male C57Bl/6 mice (**Figure 5A**). This shows that the lack of *Cyp2j5* gene in *Cyp2j5^−/−^* mice has less advantage over C57Bl/6 to block the action of Ang-II in NECA-induced concentration dependent aortic relaxation. However, Ang-II blocking action on the NECA-induced concentration dependent relaxation is stronger in male *Cyp2j5^−/−^* than the blocking action in male C57Bl/6 mice. Therefore, we conclude that inhibition or deletion of CYP-epoxygenases in mice may have stronger toxic action of Ang-II in NECA-induced concentration dependent relaxation in male *Cyp2j5^−/−^* compared to male C57Bl/6 mice, because deletion or inhibition of CYP-epoxygenases activity causes less EETs to form and involved in vasoconstriction, reduced coronary reactive hyperemic response with the association of vascular inflammation (Nayeem et al., 2008; Nayeem et al., 2009; Nayeem et al., 2010; Nayeem et al., 2011; Ponnoth et al., 2012b; Hanif et al., 2016b; Hanif et al., 2017b).

There were no significant differences observed between female *Cyp2j5^−/−^ vs*. female C57Bl/6 mice, similar to male *Cyp2j5^−/−^ vs*. male C57Bl/6 mice. Lower NECA-induced relaxation was observed in Ang-II infused female C57Bl/6/Ang-II infused female *Cyp2j5^−/−^* mice compared to non-treated female *Cyp2j5^−/−^*/female C57Bl/6 mice (**Figure 5B**). However, no significant difference in NECA-induced concentration dependent vascular response was observed between Ang-II infused female C57Bl/6 *vs.* Ang-II infused female *Cyp2j5^−/−^* mice (**Figure 5B**), and there was no significant difference in NECA-induced concentration dependent vascular response observed between Ang-II infused male C57Bl/6 *vs.* Ang-II infused female C57Bl/6 mice (**Figure 5C**). In contrast, Ang-II blocking action on the NECA-induced concentration dependent relaxation is stronger in male *Cyp2j5^−/−^ vs*. Ang-II + male C57Bl/6 mice compared to Ang-II + female *Cyp2j5^−/−^ vs*. Ang-II + C57Bl/6 female mice (**Figures 5A, B**). Also, Ang-II blocking action on the NECA-induced concentration dependent relaxation is more dominant in male *Cyp2j5^−/−^ vs*. Ang-II + female *Cyp2j5^−/−^* mice compared to Ang-II + female *Cyp2j5^−/−^ vs*. female *Cyp2j5^−/−^* mice (**Figure 5D**). This disparity between Ang-II treated male *Cyp2j5^−/−^ vs*. Ang-II treated female *Cyp2j5^−/−^* mice were prominent in NECA-induced vascular response. However, this type of difference in NECA-induced vascular response was not observed between non-treated male *vs.* female *Cyp2j5^−/−^* mice. This observation is in agreement with Ma et al. where mouse kidneys demonstrated higher *Cyp2j5* expression in male compared to female mice after puberty, and Northern analysis also revealed that *Cyp2j5* transcripts were more abundant in adult male *versus* adult female kidneys (Ma et al., 2004). However, an increased in blood pressure reported with enhanced renal (afferent arterioles) vasoconstriction with angiotensin II in female *Cyp2j5*^−/−^ compared to its respective female wild-type mice (Athirakul et al., 2008). In contrast, we demonstrated in the current study that an enhanced adenosine (NECA)-induced aortic vasoconstriction in male *vs.* female *Cyp2j5*^−/−^ mice when Ang-II infused. This difference may be due to different blood vessels (renal afferent arterioles *vs.* aorta) and different media (Ang-II alone *vs.* NECA + Ang-II). Before Ang-II treatment, there was no significant difference noticed between male *vs.* female *Cyp2j5*^−/−^ and between C57Bl/6 *vs.* *Cyp2j5*^−/−^ mice in NECA-induced aortic response. However, there was no significant difference observed in acetylcholine-concentration dependent aortic response between male *vs.* female *Cyp2j5*^−/−^ mice when Ang-II infused, but the significant difference was observed between C57Bl/6 *vs.* *Cyp2j5*^−/−^ mice in acetylcholine-concentration dependent aortic response without Ang-II treatment. Also, Burgess et al., showed that *Cyp2j5* is responsible for production of primarily 14,15- and 11,12-EETs in visceral adipose tissue (Burgess et al., 2012), and the overexpression of *Cyp2j5* and *Cyp4a* proteins were observed in *Ephx2*^−/−^ *vs.* C57Bl/6 mice with enhanced adenosine (NECA) and CGS 21680 (A_2A_ AR)-induced aortic relaxation (Nayeem et al., 2013). Any deletion or inhibition of CYP-epoxygenases activity causes less EETs to form and participate in vasoconstriction and reduced coronary reactive hyperemic response with the association of vascular inflammation (Nayeem et al., 2008; Nayeem et al., 2009; Nayeem et al., 2010; Nayeem et al., 2011; Ponnoth et al., 2012b; Hanif et al., 2016b; Hanif et al., 2017b).

NECA is an analogue of adenosine, and adenosine activates four adenosine receptors (A_1_AR, A_2A_ AR, A_2B_ AR, and A3 AR). Out of these four receptors, A_1_AR and A_3_ AR are involved in vascular contraction, whereas A_2A_ AR and A_2B_ AR are vasodilators. Previously, we have NECA concentration dependent vascular response with Ang-II treatment in both *Cyp2j5^−/−^* and C57Bl/6 mice. Now, we used CGS 21680 (A_2A_ AR agonist), DDMS (20-HETE synthesis inhibitor, dibromo-dodecenyl-methylsulfimide), and Ang-II. There was a significant difference noted between DDMS-treated male *Cyp2j5^−/−^ vs*. non-treated male *Cyp2j5^−/−^* mice, DDMS enhanced CGS 21680-concentration dependent relaxation male *Cyp2j5^−/−^ vs*. non-treated male *Cyp2j5^−/−^* mice, whereas, no significant difference in CGS 21680-concentration dependent vascular response was observed in between DDMS + Ang-II treated male *Cyp2j5^−/−^ vs*. non-treated male *Cyp2j5^−/−^* mice. In contrast, a huge difference in CGS 21680-concentration dependent vascular response was observed between DDMS + Ang-II treated male *Cyp2j5^−/−^ vs*. DDMS-treated male *Cyp2j5^−/−^* mice. Similarly, there was a significant difference observed between DDMS-treated female *Cyp2j5^−/−^ vs*. non-treated female *Cyp2j5^−/−^* mice, DDMS treatment increased CGS 21680-concentration dependent relaxation in female *Cyp2j5^−/−^ vs*. non-treated female *Cyp2j5^−/−^* mice, whereas, no significant difference in CGS 21680-concentration dependent vascular response was observed in between DDMS + Ang-II treated female *Cyp2j5^−/−^ vs*. non-treated female *Cyp2j5^−/−^* mice, except at 10^−7^ M CGS 21680. However, a highly significant difference in CGS 21680-concentration dependent vascular response was observed between DDMS + Ang-II treated female *Cyp2j5^−/−^ vs*. DDMS-treated female *Cyp2j5^−/−^* mice. These observations are in agreement with our earlier work, where A_2A_ AR (CGS 21680)-induced concentration dependent enhanced vascular (aortic) relaxation in C57Bl/6 *vs.* A_2A_ AR^−/−^ mice, enhanced relaxation was abolished with MS-PPOH (CYP-epoxygenases inhibitor) and enhanced relaxation in *Ephx2*^−/−^ (more EETs) *vs.* C57Bl/6 mice (Nayeem et al., 2008; Ponnoth et al., 2012b; Nayeem et al., 2013; Pradhan et al., 2014; Pradhan et al., 2015). Also, our current observation is in agreement with our lab reports, where, NECA- or CGS 21680-induced vascular relaxation were enhanced with the treatment of DDMS (ω-hydroxylase or 20-HETE inhibitor) or HET0016 (ω-hydroxylase or 20-HETE inhibitor) in mouse aorta or mouse mesenteric arteries with or without Ang-II treatment (Nayeem et al., 2009; Nayeem et al., 2010; Nayeem et al., 2011; Ponnoth et al., 2012a; Kunduri et al., 2013; Yadav et al., 2015; Yadav et al., 2016).

**Figure 6:**
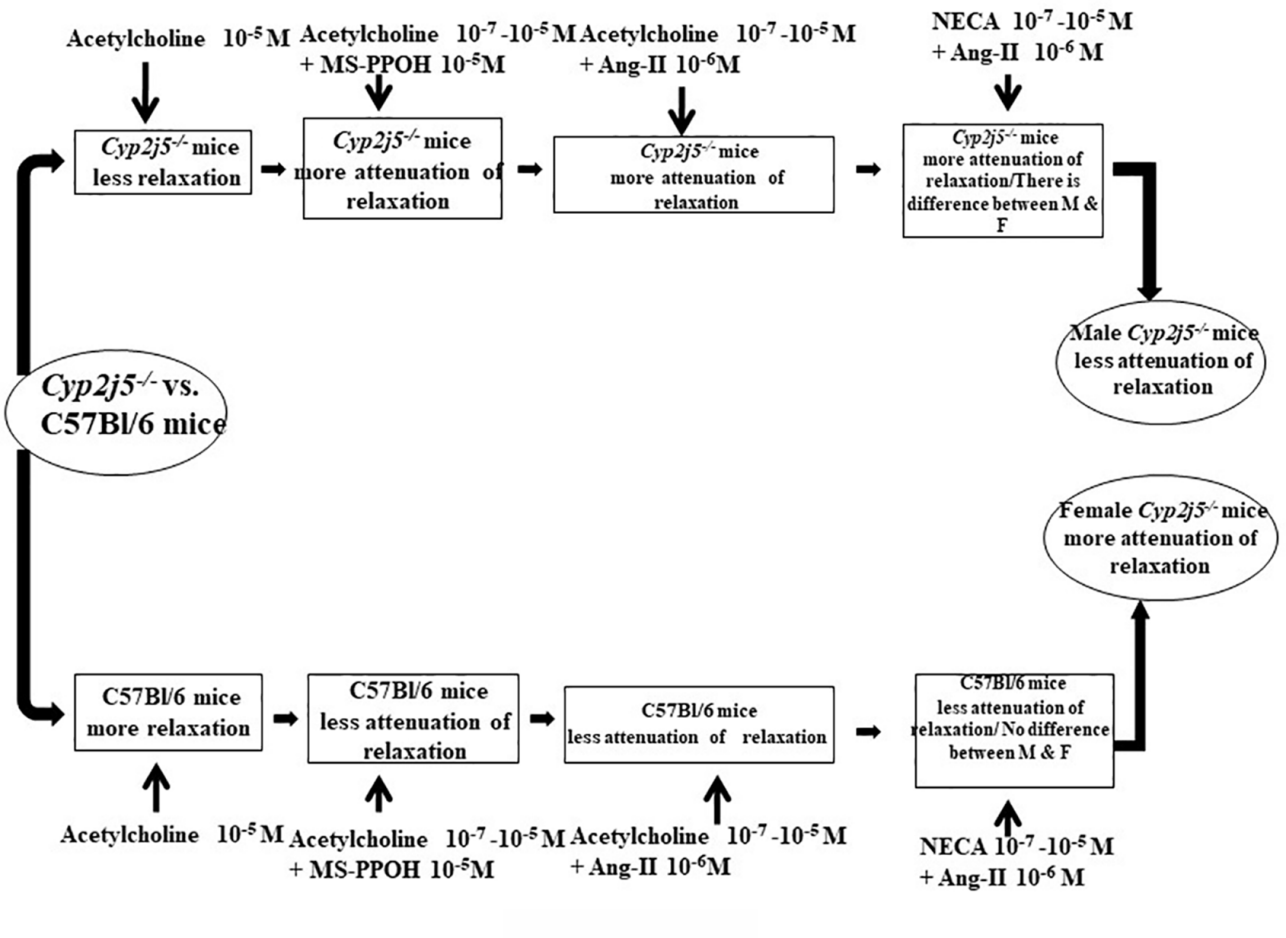
Flow diagram: comparison between *Cyp2j5^−/−^ vs*. C57Bl/6 mice treated with acetylcholine, acetylcholine + MS-PPOH, acetylcholine + angiotensin II (Ang-II), 5'-N-ethylcarboxamidoadenosine (NECA), NECA + Ang-II, and CGS 21680 + dibromo-dodecenyl-methylsulfimide (DDMS) + Ang-II.

Note: Unfortunately, we lost *Cyp2j5^−/−^* mouse colony, and this colony is not available anywhere except frozen embryos, therefore, some areas of this manuscript has some limitations. In future, we will revive this colony as the grant gets funded.

In summary, the result reported here provide new insights that inhibition or absence of *Cyp2j5* enzyme through deletion of *Cyp2j5* gene appears to be critical for acetylcholine- or NECA- or CGS 21680-induced concentration dependent vascular response while challenged by angiotensin-II or MS-PPOH (**Figure 6**). When the *Cyp2j5* gene was absent (*Cyp2j5^−/−^* mice), ACh-induced concentration dependent relaxation was significantly lower than C57Bl/6 mice. However, when the ACh-induced concentration dependent relaxation was challenged by MS-PPOH, absence of *Cyp2j5* made no significant difference in the ACh-induced concentration dependent relaxation compared to the non-treated *Cyp2j5^−/−^* mice. In contrast MS-PPOH-treated C57Bl/6 mice made a significant difference compared to non-treated C57Bl/6 mice. When the ACh-induced concentration dependent relaxation was challenged by Ang-II in *Cyp2j5^−/−^* mice, a significant difference was observed in the blocking action on ACh-induced concentration dependent relaxation compared to C57Bl/6 mice. Similarly, when the NECA-induced concentration dependent relaxation was challenged by Ang-II in male *Cyp2j5^−/−^ vs*. female *Cyp2j5^−/−^* mice and male *Cyp2j5^−/−^ vs*. male C57Bl/6 mice, a significantly huge difference in the blocking of NECA-induced concentration dependent relaxation was observed. However, NECA-induced concentration dependent vascular response itself did not make any significant difference between the *Cyp2j5^−/−^ vs*. C57Bl/6 mice. In addition, DDMS enhanced CGS 21680-induced concentration dependent relaxation in DDMS treated *Cyp2j5^−/−^ vs*. non-treated *Cyp2j5^−/−^* mice, and there was no difference was observed between DDMS + Ang-II + *Cyp2j5^−/−^ vs*. non-treated *Cyp2j5^−/−^* mice. Therefore, we conclude that inhibition or deletion of *Cyp2j5* in mice may have an increase in toxic action of both Ang-II and MS-PPOH in ACh/NECA-induced concentration dependent relaxation in *Cyp2j5^−/−^* compared to C57Bl/6 mice, and DDMS may have rescued from the toxic effect of Ang-II treatment in *Cyp2j5^−/−^* mice.

## Data Availability Statement

All datasets generated for this study are included in the article/supplementary material.

## Ethics Statement

West Virginia University Institutional Animal Care and Use Committee approved all animal care and experimentation protocols, which were in accordance with the principles and guidelines of the NIH's *Guide for the Care and Use of Laboratory Animals*.

## Author Contributions

MN: conception, design of research, performing experiments, analysis drafting and editing. SA and AH cooperated in the experimentations, reading, correction, editing and input. ME helped in correction, editing and input and DZ advised, read, corrected, edited, provided transgenic mice and input.

## Funding

This work was supported by a National Institutes of Health Grant HL-114559 to MN and a National Institute of Environmental Health Sciences Grant Z01 ES025034 to DZ.

## Conflict of Interest

The authors declare that the research was conducted in the absence of any commercial or financial relationships that could be construed as a potential conflict of interest.
